# 44 Pediatric onset systemic lupus erythematosus (p-SLE)

**DOI:** 10.1093/rheumatology/keac496.040

**Published:** 2022-10-07

**Authors:** Sheren Maher

**Affiliations:** Professor of Pediatric Rheumatology, Minia University, Minia

**Keywords:** Systemic lupus erythematosus, thrombomodulin, D-dimer

## Abstract

**Background:**

Pediatric onset Systemic lupus erythematosus (p-SLE) is multisystem autoimmune disease with disease onset before the 18th birthday. It is a highly complex disease with marked heterogeneity between patients, causing anything from mild to life-threatening disease.

**Objective:**

To assess thrombomodulin (TM) and D-dimer levels in pediatric systemic lupus erythematosus patients and their correlation to disease activity.

**Methods:**

This study included 40 children diagnosed with systemic lupus erythematosus. They were grouped according to the Systemic Lupus Erythematosus Disease Activity Index (SLEDAI) score into group (A) [Mild activity] included 20 children with SLEDAI score less than or equal to 4, group (B) [Moderate-high activity] included 20 children with SLEDAI score >4. Also, 40 apparently healthy children were included as a control group. They were age and sex matched, with the children having SLE.

**Results:**

The two studied groups had significantly higher TM level than controls, furthermore group B had significantly higher TM level than group A (*p*< 0.008 in all). The two studied groups had significantly higher frequency of abnormally high TM level than controls, furthermore group B significantly had higher frequency of abnormally high TM level than group A (*p*< 0.02 in all). SLE children had significantly higher D dimer level than controls, furthermore group B had significantly higher D dimer level than group A (*p*≤ 0.006 in all). The two studied groups had significantly higher frequency of abnormally high D dimer level than controls, furthermore group B had significantly higher frequency of abnormally high D dimer level than group A (*p*≤ 0.03 in all). TM had a significant positive correlation with SLEDAI score as r = 0.342 and *p* = 0.031. D dimer had a significant positive correlation with SLEDAI score as r = 0.42 and *p* = 0.007.

**Conclusion:**

Thrombomodulin and D dimer are potentially valuable markers of the disease activity and thrombotic tendency in p SLE patients. Thus, regular evaluation may be useful for early detection of thrombotic complications.

